# Temperature Monitoring and Material Flow Characteristics of Friction Stir Welded 2A14-t6 Aerospace Aluminum Alloy

**DOI:** 10.3390/ma12203387

**Published:** 2019-10-17

**Authors:** Tingke Wu, Fengqun Zhao, Haitao Luo, Haonan Wang, Yuxin Li

**Affiliations:** 1 School of Mechanical Engineering, Shenyang Ligong University, Shenyang 110159, China; wutingke@sia.cn (T.W.); zhaofengqun@sia.cn (F.Z.); 2 State Key Laboratory of Robotics, Shenyang Institute of Automation, Chinese Academy of Sciences, Shenyang 110016, China; 1770174@stu.neu.edu.cn (H.W.); liyuxin@stumail.neu.edu.cn (Y.L.); 3 Institutes for Robotics and Intelligent Manufacturing, Chinese Academy of Sciences, Shenyang 110016, China; 4 Institute of Mechanical Engineering and Automation, Northeastern University, Shenyang 110819, China

**Keywords:** temperature monitoring, numerical simulation, material flow model, turbulence phenomenon, welding defects

## Abstract

Aiming at the problems that the temperature in the welding area of friction stir welding (FSW) is difficult to measure and the joints are prone to defects. Hence, it is particularly important to study the material flow in the welding area and improve the welding quality. The temperature of the tool shoulder and the tool pin was monitored by the wireless temperature measuring system. The finite element model of friction stir welding was established and the welding conditions were numerically simulated. The flow law of material of the friction stir welding process was studied by numerical simulation. The material flow model was established by combining the microstructure analysis results, and the forming mechanism of the defects was analyzed. The results show that the temperature in the welding zone is the highest at 1300 rpm, and the temperature at the tool shoulder is significantly higher than that at the tool pin in the welding stage. When high-rotation speeds (HRS) are chosen, the material beneath the tool shoulder tends to be extruded into the pin stirred zone (PSZ) after flowing back to the advancing side. This will cause turbulence phenomenon in the advancing side of the joint, which will easily lead to the formation of welding defects. In the future, temperature monitoring methods and the flow model of material can be used to optimize the welding parameters.

## 1. Introduction 

Friction stir welding (FSW) has been extensively utilized to weld various aluminum alloys since its invention in 1991 [1]. Due to the advantages over traditional fusion joining techniques, such as the lack of melting, low defect and low distortion, FSW has been widely applied in the aerospace, automobile and railway industries [2,3]. It uses the high-speed rotating tool to generate heat by friction on the workpiece to promote the plastic flow of the material and realize weld formation under the forging action of the shoulder [4,5]. The traditional fusion welding process is prone to defects such as welding hot cracks, inclusions, porosity, alloy element burning, and welding residual deformation, which will significantly reduce the welding quality. Compared with fusion welding, friction stir welding has the advantages of solid-state connection, lower production cost, and excellent joint performance in aluminum alloy welding [6,7,8]. If the welding parameters are not properly controlled, it will easily lead to changes in physical processes such as material heat generation, plastic flow state and even lead to welding defects [9,10].

**Figure 1:**
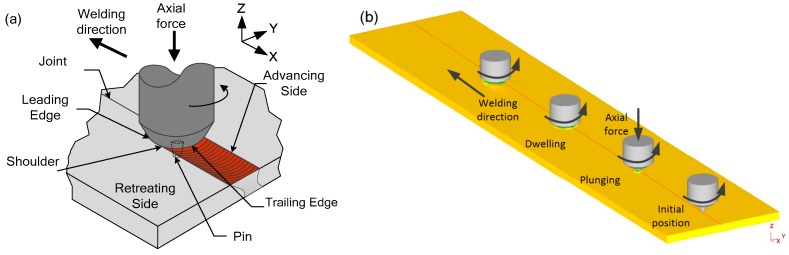
Schematic diagram of welding process. (**a**) Schematic diagram of friction stir welding area; (**b**) different stages of welding process.

As shown in Figure 1a, the tool pin is mechanically inserted into the welding of the workpiece during friction stir welding. The high-speed rotating tool plastically softens the material in the welding zone. When the stirring tool reaches the predetermined welding depth, it is welded along the weld direction under the action of feed force. The highly plastic material behind the tool forms a compact weld joint under the forging force of the shoulder [11]. As shown in Figure 1b, a part of the material is extruded above the workpiece in the initial plunging stage. After the tool is plunged to a certain amount, it enters the dwelling phase, and a small amount of flash is easily generated during welding due to the extrusion of the shoulder [12,13,14].

Since the invention of FSW, some researchers have tried to understand the material flow in the welded zone through experiments. Some methods, such as tracer technique by marker [15,16,17,18], FSW of different metallic materials [19,20], and microstructural observations [21,22], have been successfully used to study the visualization of material flow. However, the information obtained through a single experiment is very limited. In order to fully understand the material flow behavior in the FSW, a large number of the welding tests are required, which is time-consuming and exhausting. Schneider [23] proposed two flow paths to determine FSW region. A tungsten wire with a diameter of 0.0025 mm was used to study the visualization process of material flow.

Compared with the experimental method, numerical simulation is a more convenient and effective method to study FSW. The visualization of material flow, temperature field, stress and strain during the whole FSW process can be easily obtained by numerical method. The numerical simulation technology can avoid many repeating experiments and save lots of energy. For these reasons, different modeling techniques, such as computational fluid dynamics (CFD) [24], arbitrary Lagrangian Eulerian formulation (ALE) [25], coupled Euler-Lagrangian method (CEL) [26,27] and the smoothed particle hydrodynamics method (SPH) [28], have been proposed to simulate the FSW process.

Guerdoux [29] proposed an arbitrary Lagrangian-Eulerian formulation (ALE) to calculate the material flow and temperature evolution in three stages of friction stir welding (FSW). According to his research, the mesh velocity was derived from the domain boundary evolution and an adaptive refinement criterion provided by error estimation, so it can simulate the unstable phase of FSW process. Schmidt [25] developed a fully coupled thermomechanical three-dimensional finite element model using arbitrary Lagrange-Eulerian formulation and Johnson-Cook material law. The model explains compressibility by including elastic response of aluminum matrix. The main purpose of the model is to analyze the main conditions for filling the cavity behind the tool.

Fraser [30] described a smoothed particle hydrodynamics method that can automatically evaluate the presence and severity of defects in welded zone. It can be used to determine the process parameters of the numerical optimization process, while minimizing the defects in the weld zone. Hossfeld [31] used coupled Euler-Lagrange (CEL) method to model the entire FSW process within a continuous model. The model can not only simulate the welding of two plates with real geometric shapes, but also simulate the formation of burrs and internal voids. The results of temperature field, surface and weld formation and process force are accurately displayed and verified.

Liu et al. [32] analyzed the heat input process of the welding process and proposes to control the welding parameters to change the heat input to reduce the influence of heat caused in different welding areas. Kadian et al. [33] studied the effects of tool pins with seven different geometric shapes on material flow. The heat loss of backplate and the convective heat loss are also considered in the simulation. The analysis is based on the established finite volume method and takes into account the transient laminar flow of the material. The material mixing effect of tool with conical shape is better than that of flat pin shape. Simar et al. [34] observed the effect of welding heat input on microstructure and mechanical properties of welds.

The above methods all reflect the friction stir welding process to varying degrees. In this study, Deform-3D software was selected to simulate the friction stir welding process. The software not only has the function of effective mesh re-division, but also has a unique point tracking method, which is helpful to simulate material flow and visualize deformation patterns. Through experiments and numerical simulation to study the flow law of material, the establishment of material flow model will increase the understanding of welding conditions, so there are still many problems to be studied.

Although there are some differences between the approximation method and the actual welding temperature. However, the temperature of the welding area can be approached to the greatest extent. At the same time, the temperature is monitored by the same position of the tool. The temperature under different welding parameters have the same temperature measurement conditions. The temperature measurement results have repeatability and effective reference. Hence, the method of temperature measurement is reasonable and feasible.

To sum up, the change of welding parameters will change the material flow in the welding zone. The temperature difference under different welding parameters can be studied by real-time monitoring of the temperature in the welding zone. The flow law of materials was studied by finite element model, and the results of finite element analysis were verified by microstructure analysis. Based on the above analysis, a material flow model is proposed to study the formation mechanism of welding defects and to analyze the formation process of turbulence in the defect area. In the future, welding parameters can be optimized by combining temperature measurement method with material flow model, which will effectively guide the improvement of welding parameters.

## 2. Finite Element Model

### 2.1. Geometric Model and Boundary Conditions

The tool material is W6, and the workpiece is 2A14-t6 aluminum alloy. The shoulder diameter of the tool is 16.3 mm, the maximum diameter of the tool is 8.15 mm, and the length of the tool is 5.65 mm. Due to the large plastic deformation of the material during the welding process, the mesh adaptive re-division technique is used to control the mesh distortion in the simulation process. The total number of workpiece meshes is 72,451. The total number of tool tetrahedral meshes is 36,283. In the simulation process, since the material strength of tool is much higher than the workpiece material, the FSW tool is defined as a rigid body, and the workpiece is defined as a rigid plastic body. At the same time, in order to simplify the model, the heat transfer coefficient is used to offset the temperature transfer of the back plate. The degree of freedom of the bottom surface and the side surface of the workpiece was fully constrained. The thermal characteristic parameters of tools and workpieces are shown in Table 1.

Deform-3D software uses adaptive mesh generation (AMG) technology. Its purpose is to ensure that unqualified meshes in the process of finite element analysis can be re-divided in real time. There are mapping method, filling method, topological analysis method, octree method, structural analysis method and rotation translation method for mesh re-division. Delaunary technique is used to generate triangular elements and tetrahedron elements, and adaptive mesh refinement can be realized [35].

### 2.2. Material Model

The appropriate principles of the material model have an essential impact on the correctness of the numerical simulation results. The material changes from a solid state to a viscous state, so it is necessary to define a wide range of flow stress values. Equation (1) shows that flow stress is defined as a function of strain, strain rate, and temperature.

**Figure 2:**
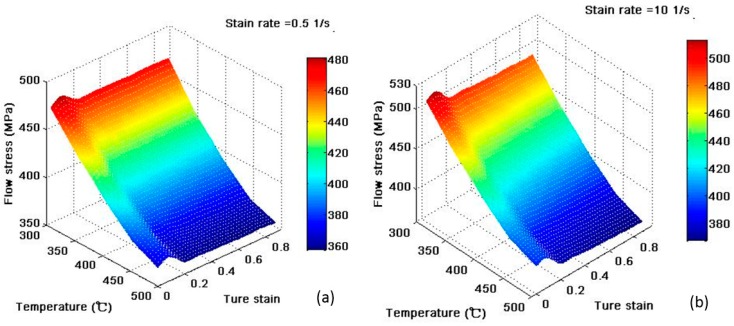
Flow stress curve of the 2A14 aluminum alloy. (**a**) The flow stress curve at strain rate of 0.5; (**b**) The flow stress curve at strain rate of 10.

In the FSW process, the material undergoes large deformation accompanied by high temperature and high strain rate. Hence, in order to capture the correct behavior of the material, it is important to define the flow stress of the material as a function of temperature, strain and strain rate. The workpiece is defined as a rigid visco-plastic material, but the elastic deformation of the material is neglected to achieve better convergence. This is a valid assumption because the effective strain in the range of 6–80 [36] is considered as the offset method of 0.002 in to calculate the yield point. Hence, the flow stress values for a wide range of strain, temperature and strain rate are defined [37].

(1) $$\bar{\sigma }=\bar{\sigma }(\varepsilon ,\dot{\bar{\varepsilon }},T)$$

where, $\bar{\sigma }$ is flow stress, ε is the ture strain, $\dot{\bar{\varepsilon }}$ is the strain rate and T is temperature. The flow stress is temperature-dependent. Figure 2 shows the flow stress curve of the 2A14 aluminum alloy. The flow stress data is imported into the numerical model. It can be seen from Figure 2 that the material is sensitive to temperature at high strain rates, and the flow stress decreases with increasing temperature. This is mainly because the thermal vibration energy of the crystal lattice is increased at a high temperature, and the external force required for the dislocation motion is reduced.

### 2.3. Thermal Model

In FSW, heat is generated due to friction and plastic deformation between tools and materials. As shown in Equation (2), inelastic thermal coefficient is defined to include heat generated due to plastic deformation [38].

(1) $$\dot{q}={\dot{q}}_{f}+{\dot{q}}_{p}$$

where, $\dot{q}$ is the total heat generated by the friction stir welding process, ${\dot{q}}_{f}$ is the frictional heat generation, and ${\dot{q}}_{p}$ is the heat generated by the plastic deformation of the material.

(2) $${\dot{q}}_{p}=\eta \bar{\sigma }\times \dot{\varepsilon }$$

where, η is the inelastic heat coefficient, which is 0.9 in this paper [39]. The temperature distribution is determined by Fourier’s law of the heat conduction equation, as shown in Equation (4).

(3) $$k\Delta^{2}T+\dot{q}=\rho c_{p}\frac{\partial T}{\partial t}$$

where, k is the thermal conductivity of the material, ρ is the density of the material, and $c_{p}$ is the heat capacity per unit mass of the material. The analysis of heat transfer was considered in the numerical simulation process.

### 2.4. Friction Model

The contact conditions at the interface between the tool and the workpiece are very complicated, and there is not much experimental evidence to show the friction conditions exactly. Researchers used different friction boundary conditions to simulate FSW simulation. A few people have used Coulomb’s law of friction [40], and some people think that the coefficient of friction is a function of pressure and slip rate [41]. Other people have used adhesive friction conditions [42]. In this simulation, the adhesion friction condition is assumed according to Equation (5). The adhesion condition is selected because it is suitable for most forming operations, and the material state in FSW can be associated with such operations [43].

(4) $$\bar{\tau }=k\tau_{\max}$$

Where $\bar{\tau }$ is the contact pressure at the interface between the tool and the workpiece. $\tau_{\max}$ is the shear yield strength of the material. According to von Mises yield criterion, it is 0.577 times as the yield strength of the material. k is the shear coefficient. According to Rahul Jain’s research [38], when the shear coefficient is selected to be 0.4, the simulated data can be well matched with the experimental data.

### 2.5. Governing equations

The finite element model uses a rigid viscoplastic model with von Mises yield criterion [35]. The finite element formula of rigid viscoplastic material is based on the variational principle. The allowable velocity A should meet the conditions of compatibility and incompressibility, as shown in formula (1).

(6) $$\eta =\int_{V}E({\dot{\varepsilon }}_{ij})dV-\int_{S_{F}}F_{i}u_{i}dS$$

where, $F_{i}$, V,$S_{F}$ and $E({\dot{\varepsilon }}_{ij})$ are the surface traction force, workpiece volume, stress surface and plastic deformation power function. The incompressibility constraint on the allowable velocity field is eliminated by adding a penalty function for incompressibility [36]. However, the actual velocity field can be determined by the stable value of the change equation, which is expressed as:

(7) $$\delta \eta =\int_{V}\bar{\sigma }\delta \dot{\bar{\varepsilon }}dV+\lambda \int_{V}{\dot{\varepsilon }}_{V}\delta {\dot{\varepsilon }}_{V}dV-\int_{S_{F}}F_{i}\delta u_{i}dS$$

where ${\dot{\varepsilon }}_{V}$ is the basic equation of Lagrange finite element method, ${\dot{\varepsilon }}_{V}$ is the volume strain rate, $\delta \dot{\bar{\varepsilon }}$ and $\delta {\dot{\bar{\varepsilon }}}_{V}$ are the strain rate changes derived from $\delta u_{i}$, and λ is the penalty factor for the volume incompressible condition.

### 2.6. Dislocation Density Model

The plastic deformation of metal originates from dislocation movement of material. So the dislocation density changes continuously during friction stir welding, and the material flow changes based on this model. This model is the internal model of DEFORM-3D software [44]. The dislocation density is expressed as follows:

(8) $$d\rho_{i}=(h-\dot{r}\rho_{i})d\varepsilon$$

(9) $$h=h_{0}{\left(\frac{\dot{\bar{\varepsilon }}}{{\dot{\varepsilon }}_{0}}\right)}^{m}\cdot \exp\left(\frac{mQ}{RT}\right)$$

(10) $$\dot{r}={\dot{r}}_{0}{\left(\frac{\dot{\bar{\varepsilon }}}{{\dot{\varepsilon }}_{0}}\right)}^{-m}\cdot \exp\left(\frac{-mQ}{RT}\right)$$

where $\rho_{i}$ is the dislocation density, h is the height of the action range of the dislocation stress field, $\dot{r}$ is the radius of the action range of the dislocation stress field, m is the rate sensitivity, and ${\dot{\varepsilon }}_{0}$ is the non-dynamic strain rate.

## 3. Test and Monitoring Methods

### 3.1. Test Process

The test used 2A14-t6 aluminum alloy plate with the same size and thickness as the numerical simulation process. 2A14-t6 aluminum alloy is a high-strength aviation aluminum alloy, and its internal material composition is shown in Table 2 [45]. The tool is of concave shoulder structure, the tool pin is of conical thread structure, the diameter of the shoulder of the tool is 16.3 mm, the concave angle of the shoulder is 10°, the cone angle of the tool pin is 15°, and the length of the tool pin is 5.65 mm. The spiral direction of the tool pin used in this study is the same as the rotation direction of the spindle. The welding process is completed on a self-developed high-precision friction stir welding robot [46]. The welding process is set to a stable welding speed of 100 mm/min, the tool shoulder is plunged into the workpiece surface by 0.25 mm, the spindle inclination is 2.5°. After the tool is pressed down to a certain amount, it will rotate for 10 s. The welding process is consistent with the parameter setting of the simulation process.

### 3.2. Temperature Monitoring Method in the Welding Zone

In order to study the welding temperature of the welding area, the temperature of the shoulder and the tool pin are monitored in real time through a wireless temperature measuring system (Shenyang Institute of Automation, Chinese Academy of Sciences, Shenyang, Liaoning, China). The temperature in the welding zone rises rapidly due to the high-speed stirring action between the tool and the material in the welding zone. The plastic flow of materials occurs in the welding area. However, it is difficult to measure the temperature of the welding area by contact method. Although some scholars use optical temperature meter and other non-contact methods to measure the temperature of the welding area [47]. The non-contact temperature measurement method is difficult to reach the expected target due to the influence of environment and reflection of metal surface.

**Figure 3:**
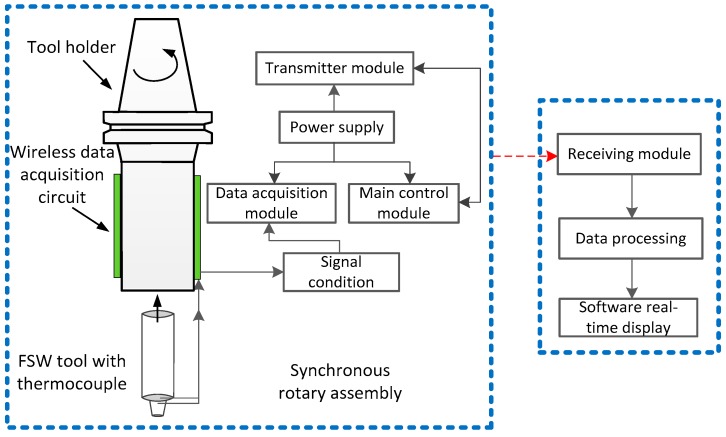
Schematic diagram of the main components of the FSW wireless temperature measurement system.

The wireless temperature measurement system mainly includes a data acquisition module, a main control module, a power supply module and a transmission module. The specific workflow is shown in Figure 3. The data acquisition module is responsible for the data acquisition of thermocouples and converting the collected analog signals into high-precision digital signals. The main control module is responsible for preliminary data processing, including positive and negative value processing, transmission bit selection and other operations. The power module is responsible for the power supply of the entire system, and the main control module transmits the processed data to the transmission module through the serial port. The data processing part is connected to the upper computer through the receiving module. The upper computer collects data through the serial port, and the acquisition software processes the data in real time and displays the temperature curves.

**Figure 4:**
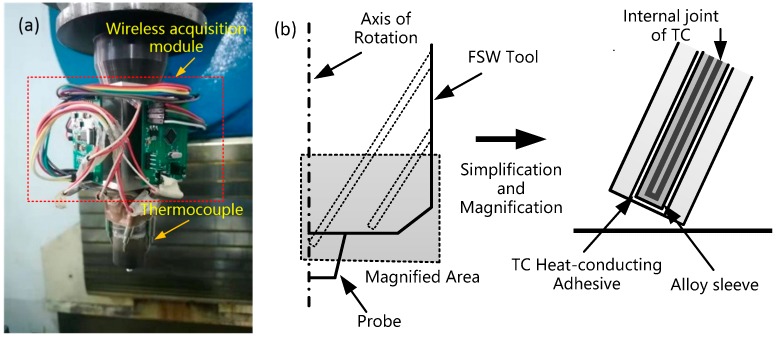
Wireless temperature measurement system. (**a**) Assembly of wireless temperature measurement system; (**b**) thermocouple temperature monitoring diagram.

Hence, a wireless temperature measurement system was designed, as shown in Figure 4a. Shoulder and tool pin are the main action positions in the welding process, so the temperature test of the tool pin and shoulder will reflect the temperature of the welding area to a great extent. In order to prevent the structural damage of the tool pin and the tool shoulder, and to ensure that the temperature measurement results are as close as possible to the temperature of the welding zone, the temperature was tested at 4 mm above the tool pin and 1.2 mm above the shoulder.

The testing principle of thermocouple is shown in Figure 4b. Thermocouple is obliquely embedded in the mounting hole. The temperature measuring principle of thermocouple is based on thermoelectric effect. The inside of the thermocouple is a closed loop composed of conductors of two different materials, and the outer layer is an alloy protective sleeve.

One thermocouple is set at the upper shoulder of the FSW tool and is defined as thermocouple (TC) point 1. In order to verify the accuracy of the test, three thermocouples are set at the tool pin, the vertical distance between each thermocouple is 4 mm, and the thermocouples are distributed in a 120 degree angle spiral rise. The main reason for the equidistant distribution of thermocouples on the axis of the tool pin is to complete the display of temperature gradient. The position distribution of 120 degree angle is to prevent interference between thermocouples. At the same time, the gradient can also verify the accuracy of the test. For convenience, the thermocouple at the lowest end of the tool pin is defined as thermocouple point 2, the thermocouple at the highest end of the tool pin is defined as thermocouple point 4. The position distribution of thermocouple 1 and 2 is set at 30 degree angle. Thermocouple point 3 is defined at the middle position between TC point 2 and TC point 4. The thermocouple of shoulder is inclined to the tool axis at 48 degree angle, and the thermocouple at the tool pin is distributed to the tool axis at 52 degree angle. In order to improve the test accuracy and ease of installation, the thermocouple 1 and the thermocouple 2 are distributed at 60°.

### 3.3. Workpiece Temperature Test Method

**Figure 5:**
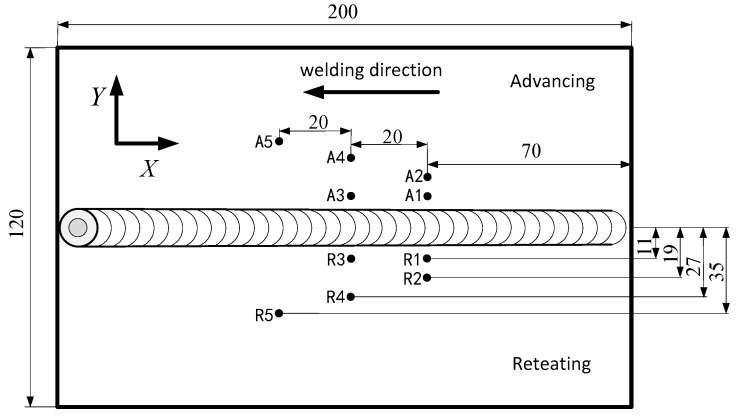
Temperature measurement point distribution on the workpiece.

In order to study the temperature distribution on the workpiece during welding, the temperature of workpiece was monitored. During the welding process, the thermocouples are used to synchronously collect the temperature of the measuring points, and the first set of measuring points is set at 80 mm from the initial welding position, because the temperature near the initial position did not reach a steady state during the welding process, while the temperature also reached a relatively steady state value with the stable welding seam formation. Specific temperature measurement points are shown in Figure 5. Thermocouples on the advancing side are defined as A1–A5, thermocouples on the retreating side are defined as R1–R5, and temperature measuring points are symmetrically distributed on both sides of the joint.

## 4. Results and discussion

### 4.1. Temperature Monitoring Results in the Welding Zone

In the process of friction stir welding, the spindle speed has a great impact on the welding temperature, while the welding temperature also has an impact on the material flow. Hence, it is important to study the material flow in the welding process by measuring the temperature in the welding zone. In this study, the temperature difference caused by the spindle speed was monitored, aiming at studying the flow law of material and understanding the influence of process parameters on welding quality.

**Figure 6:**
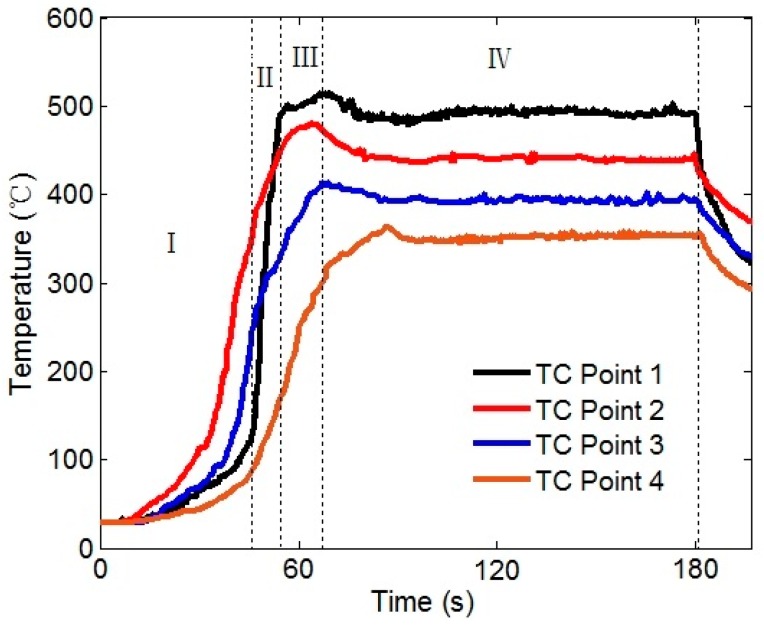
Temperature test results of the welding area (900 rpm spindle speed and 100 mm/min welding speed).

In this study, the wireless temperature measurement system accurately captures the dynamic characteristics of temperature in the welding process. Since thermocouples are extremely close to the tool pin and the shoulder, the temperature measurement results at 900 rpm are shown in Figure 6, where TC point 1 is the temperature measurement point at the shoulder and TC point 2 is the temperature measurement point at the tool pin. In the initial stage of the plunging, the material in the contact area is plastically softened due to the tool pin just contacting with the material. The friction at the interface intensifies due to the high-speed rotation of the spindle, and the temperature at the tool pin increases rapidly. Hence, the temperature at the thermocouple point 2 rises faster in this stage. It can be seen from the curve of TC point 1 that both the first and second areas belong to the plunging stage. The second area is the contact of the shoulder with the workpiece, and the temperature at the shoulder rises rapidly and exceeds the temperature of the tool pin area. The third zone is a dwelling phase in which the temperature rises extremely slowly or even at a certain temperature. The fourth zone enters the welding phase, and the temperature of the welded zone in the welding phase undergoes a cooling process, and then the temperature enters a dynamic stabilization phase.

Through the thermocouple points 2, 3 and 4 of the tool pin, it can be seen that the temperature of the tool pin is always lower than that of the shoulder during welding process, which is consistent with the theory that more heat is generated at the interface of the tool shoulder [13]. At the same time, the temperature gradient of different measuring points at the tool pin has certain regularity, which also shows the correctness of the temperature measurement results.

**Figure 7:**
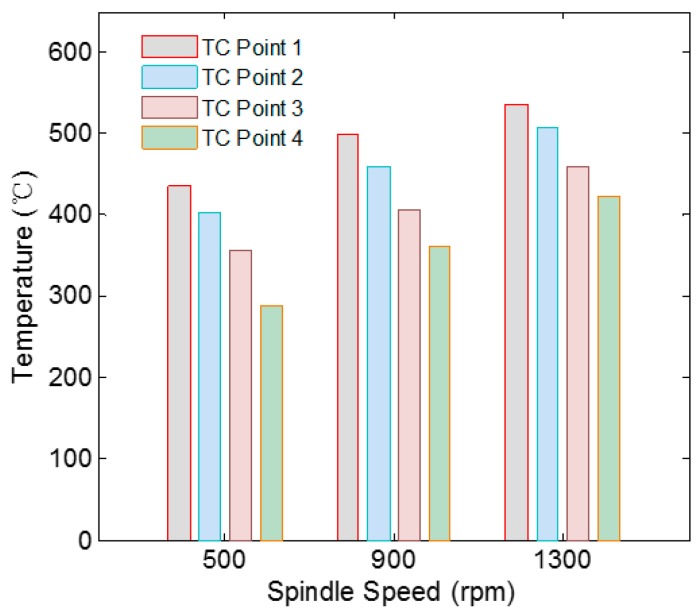
Temperature distribution at different spindle speeds.

The temperature of tool shoulder and tool pin under different rotational speeds was monitored. The test results are shown in Figure 7. With the increase of rotational speed, the temperature of welding zone keeps rising. When the rotational speed of spindle rises from 500 rpm to 900 rpm, the average temperature of welding zone rises by 60.3 °C. When the rotational speed of spindle rises from 900 rpm to 1300 rpm, the average temperature of welding zone rises by 42.5 °C. For higher temperature, the flow stress of metal in welding zone decreases, which leads to the reduction of spindle torque. The friction between tool and workpiece is weakened, which reduces the heat generation and leads to the decrease of the rate of temperature rise [18]. The change of spindle speed during welding will lead to the change of material flow. Hence, the flow law of material can be fully studied by monitoring the welding process temperature combined with the welded joint test.

### 4.2. Temperature Test Results of the Workpiece

**Figure 8:**
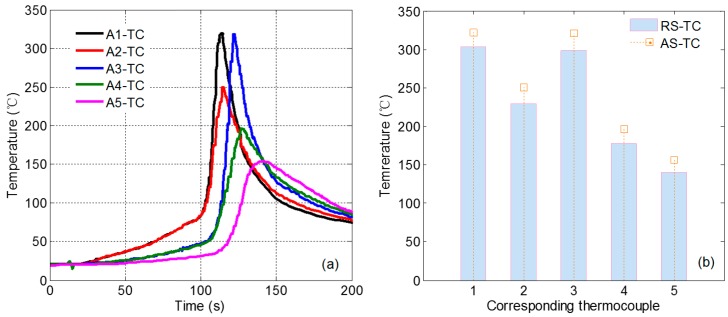
Monitoring data of workpiece temperature. (**a**) Temperature curve on the advancing side; (**b**) temperature comparison on the advancing side and the retreating side.

Figure 8a shows that the initial welding process A1 and A2 have the same temperature rise trajectory. When the tool passes the temperature measurement point, the temperature rises rapidly, and the temperature at A1 reaches 322 °C. Since A2 is far away from the weld, the temperature is relatively low. The temperature at A3 is 321 °C, and the temperature difference between A1 and A3 is relatively small, indicating that the welding process temperature is relatively stable and the accuracy of the test results. At the same time, the temperature curves of A2, A4 and A5 shows a good temperature gradient. The temperature dropped to 154 °C at the A5 measuring point. It is observed that the temperature at this measuring point decreases slowly compared with other measuring points. The reason may be that it is far away from the welding seam, and the temperature at this point reaches a relative dynamic balance due to the long-term temperature accumulation in the welding process. The temperature in this area decreases relatively slowly during the welding process.

Figure 8b shows the temperature comparison between the advancing side and the retreating side, wherein the temperature on the retreating side is lower than the temperature on the advancing side as a whole, with an average of 19 °C. However, the difference between the temperature at R4 and R5 and the temperature on the advancing side is obviously smaller. Combined with the situation of A5 point on the advancing side, it can be judged that the temperature measurement point at a certain distance from the weld is in a relatively dynamic stable phase, which is similar to the situation on the advancing side. The temperature on the advancing side is higher than that on the retreating side, which indicates the asymmetry of friction stir welding process. The difference in material flow between the advancing side and the retreating side is also closely related to temperature.

### 4.3. Axial Force and Torque

**Figure 9:**
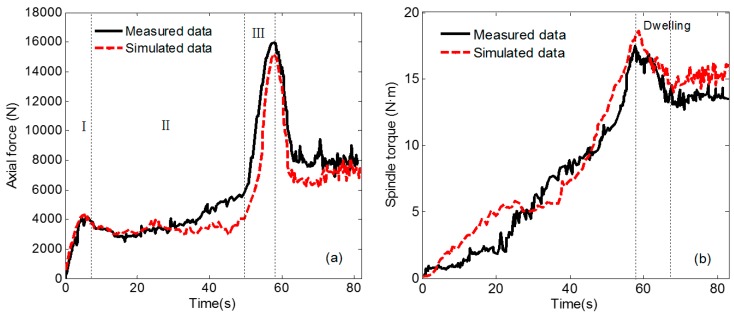
Comparison of axial force and torque data (Spindle Speed 900 rpm). (**a**) Validation of axial force; (**b**) validation of spindle torque.

Mechanics research is also an important aspect of FSW. It directly shows the loading situation of FSW tools in the welding process. As different materials flow will produce different flow resistance, the mechanical results also reflect the material flow situation in the welding process to a certain extent, which is a beneficial supplement to temperature testing. Figure 9a shows the evolution history of axial force during welding. It can be seen from the curve that the tool pin has just come into contact with the workpiece, and some materials are squeezed out by plastic shearing. After the axial force reaches the peak value in the first stage, the axial force decreases. As shown in the temperature curve in Figure 6, the temperature rises rapidly in this stage, the material around the tool pin undergoes plastic softening, and the external force of the workpiece plunging into the material increases slowly [38]. The third stage is the contact between the tool shoulder and the material. The contact area between the tool shoulder and the workpiece increases rapidly and the corresponding axial force increases significantly. When the FSW tool is plunged down to the specified depth, the FSW tool will perform dwelling rotation. At this time, the tool will suffer less resistance and the axial force will decrease. In the welding process, the axial force increases again, and finally it is in a stable state.

The change in the spindle torque curve compared to the axial force is gradual. The spindle torque and the axial force have a similar change trend as a whole. As shown in Figure 9b, the spindle torque is relatively small at the beginning of the plunging stage. As the contact area between the tool pin and the material increases gradually, the spindle torque also increases continuously. During the dwelling phase of the tool, the torque gradually decreases. The torque change in welding phase is relatively gentle. There is a certain error between the simulated data and the experimental data. The simulated curve changes relatively gently in the plunging stage, but the overall numerical value and trend are consistent with the experimental data. Hence, the finite element model is also verified to be relatively accurate.

**Figure 10:**
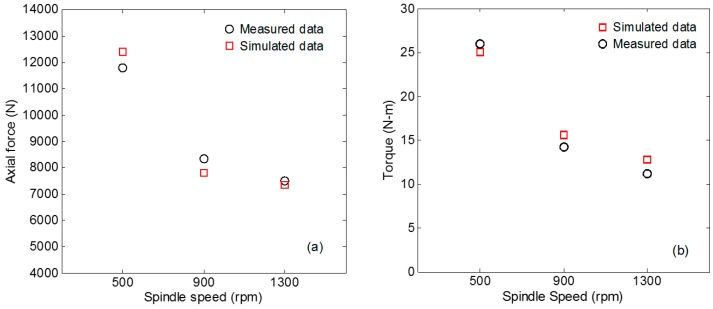
Data comparison verification of axial force and torque. (**a**) Comparison of axial force data; (**b**) comparison of spindle torque data.

Figure 10 shows the force and spindle torque at three different rotational speeds. As the rotating speed of the spindle increases, the friction between the FSW tool and the workpiece interface is higher in unit time, the heat generated in the welding area increases, the plastic softening degree of the material increases, and simultaneously the force and the spindle torque decrease with the increase of the rotating speed. When the spindle speed rises from 500 rpm to 1300 rpm, the axial force decreases by 4163 N and the torque decreases by 15.2 N-m. It was observed that when the spindle speed was increased from 900 rpm to 1300 rpm, the reduction of axial force and torque became smaller. Combining with the temperature data of Figure 10b, the increase of temperature in this process is also slowed down, and the plastic softening of material has reached a certain state, at which time the resistance of FSW tool changes slightly.

### 4.4. Numerical Simulation of Material Flow

**Figure 11:**
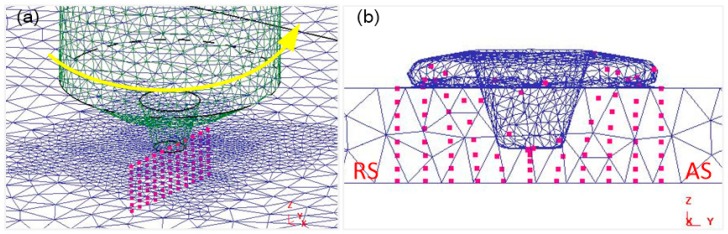
Tracking particles and the plunging process. (**a**) The overall distribution of particles; (**b**) cross section distribution perpendicular to welding direction.

In the initial plunging stage, the material is squeezed and overflowed by the tool. With the increasing depth of tool pin plunging, the degree of material plastic softening increases, and the plastic flow occurs in the area of tool pin plunging. As shown in Figure 11, it can be seen that the tracking particles spiral upward along the tool pin during the plunging stage. Eleven groups of tracking particles were set up, and the distance of each group was 1.7 mm. 

**Figure 12:**
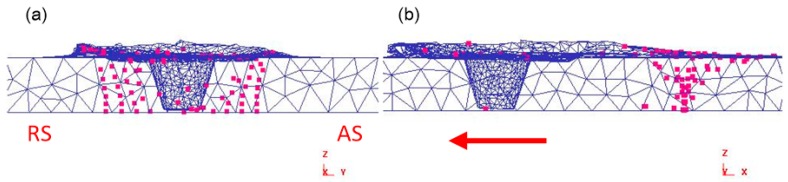
Distribution of tracking particles after welding. (**a**) Cross section distribution perpendicular to welding direction; (**b**) cross section distribution in welding direction.

Figure 12 shows the particle distribution after welding, and it can be seen that the material flow on the advancing side is higher than that on the retreating side. After welding, there are relatively few particles on the upper layer of the advancing side, and the flow trend of the particles is similar to the shape of the tool pin and presents a cone shape. Some upper particles are extruded to the lower part of the shoulder, and some particles in the thermo-mechanically affected zone (TMAZ) are dragged to the welding nugget area. At the same time, the flow degree of material below the welding zone is smaller than the particles above the welding zone.

**Figure 13:**
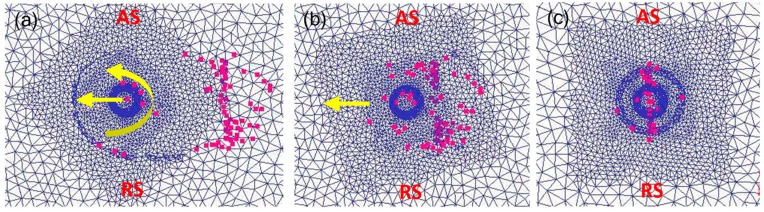
Tracking the distribution of particles. (**a**) The plunging stage; (**b**) particle flow; (**c**) welding end.

Figure 13c shows that there is a certain material flow during the plunging stage. When the tool is pressed down to a certain amount, the flowing particles are uniformly distributed at the shoulder interface. After the welding was finished, the particles were rotated around the tool and finally deposited behind the weld. Figure 13a shows that during the welding process, a small amount of particles adhere to the shoulder and the tool pin for rotating movement, and several particles adhered to the shoulder finally stay on the back side of the weld. At the same time, Jain [48] also found a similar process. The results show that the material is rotated for a long time by the tool pin in the plunging stage, the particle flow distribution is relatively uniform, and the flow degree of material on the advancing side is higher than that on the retreating side in the welding stage.

### 4.5. Effective Strain in the Welding Zone

**Figure 14:**
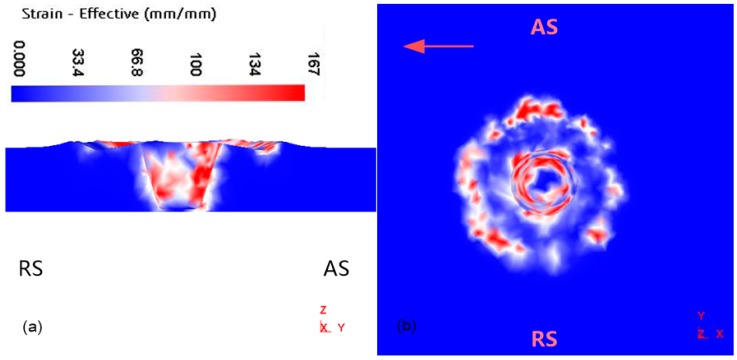
Effective strain distribution of spindle speed 900 rpm. (**a**) Cross section distribution perpendicular to welding direction; (**b**) the surface distribution of welded joints.

During the welding process, the plastic deformation of material occurs in the shoulder and the tool pin area. At the same time, due to the concave structure of the tool shoulder, the outer profile of the shoulder has a significant effect on the material, this feature can also be shown in Figure 14. The effective strain on the advancing side is significantly higher than that on the retreating side. However, the effective strain reflects the action strength between the FSW tool and the material, thus indicating that the flow strength of material on the advancing side is greater than that on the retreating side. The positive velocity component of the advancing side has more shearing action relative to the other side. The gradient of the speed difference on both sides of the weld results in asymmetry in the friction stir welding process. Hence, the effective strain further reflects the plastic deformation and the flow law of material.

### 4.6. Material Flow Model

**Figure 15:**
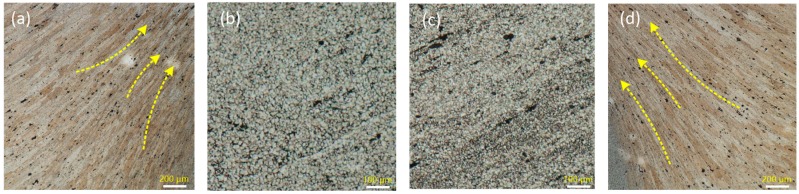
The microstructure of deformation zone of joint. (**a**) The microstructure on the advancing side; (**b**) shoulder stirring area (SSZ); (**c**) pin stirring area (PSZ); (**d**) the microstructure on the retreating side.

Figure 15b shows the microstructure of the shoulder stirring zone and Figure 15c shows the microstructure of the pin stirring zone. There are obvious differences in microstructure between the shoulder stirring area and the pin stirring area. The flow trend of the material can be clearly seen from the microstructure formation. However, it can be seen from Figure 15a that the material in TMAZ region has an upward flow trend due to the rotation of the tool pin. At the same time, some of the material is squeezed into the weld nugget region, which is also found in the simulation. A similar flow trend is also shown on the advancing side, as shown in Figure 15d. However, it can be observed that the flow slope of material on the advancing side is larger, which also verifies that the material flow on the advancing side is more intense.

**Figure 16:**
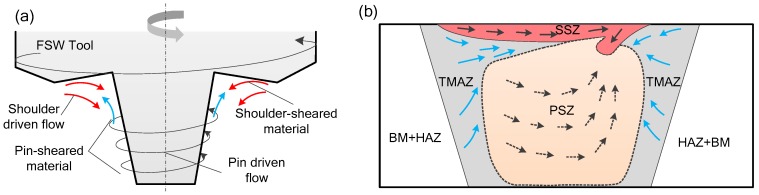
Plastic flow model of material in FSW process. (**a**) Schematic diagram of material plastic flow near FSW tool; (**b**) flow pattern of the joint deformation zone.

A material flow model is proposed based on numerical simulation results and microstructure flow trends. As shown in Figure 16, the material flow caused by the tool during the friction stir welding process can be divided into two parts, namely the shoulder driven flow and the tool pin driven flow. Generally, the upper part of the pin-sheared material tends to be squeezed into the concave area of the shoulder, and this part of material and shoulder-sheared material together fill the hole behind the welding tool by the shoulder driving action, so the material flow pattern in this area is called shoulder driven flow. The rest of the pin-sheared material that has not been extruded to the concave shoulder undergoes helical upward movement with the direction of rotation. This process was found in the numerical simulation, and this flow pattern is called pin-driven flow. The shoulder-driven flow area is defined as the shoulder stirring area (SSZ), and the pin-driven flow area is called the pin stirring area (PSZ).

**Figure 17:**
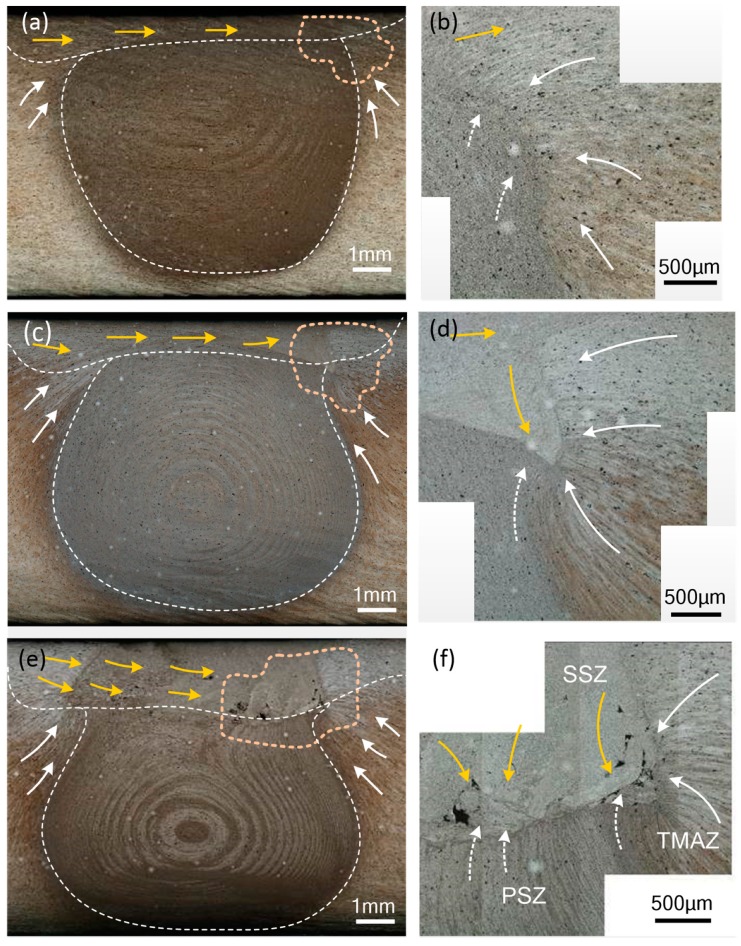
The joint cross section and material flow pattern at different rotating speeds. (**a**,**c**,**e**) are the joint cross sections at 500 rpm, 900 rpm and 1300 rpm respectively; (**b**,**d**,**f**) are material flow patterns corresponding to (**a**,**c**,**e**).

As shown in Figure 17, the cross section of the welded joint has a clear contour dividing line. When the spindle speed is increased from 500 rpm to 1300 rpm, the volume of the shoulder driven material increases, and the depth of the shoulder stirring zone gradually increases, which means that the increase of the rotational speed increases the stirring effect of the shoulder. The increase in the rotational speed causes the area of the material intersection on the advancing side to gradually become larger, which is the material intersection of the shoulder stirring area, the pin stirring area and the thermo-mechanically influence area. When the rotating speed increases, the pin stirring area expands to both sides, and the material in the thermo-mechanically affected area is dragged into the pin stirring area, forming a typical "onion ring" [49]. The increase of the rotating speed increases the downward movement trend of the shoulder driven material and the upward movement trend of the pin driven material. When the rotating speed increases to 1300 rpm, the turbulence phenomenon on the advancing side causes the joint to have hole defects.

**Figure 18:**
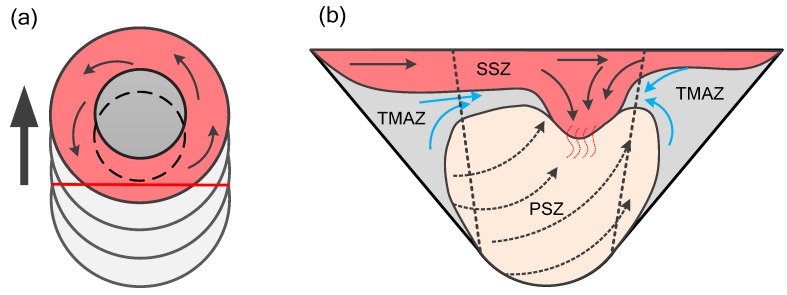
Schematic diagram of turbulent defect formation. (**a**) The surface of the welded joint; (**b**) the cross section of the welded joint.

As shown in Figure 18, during the welding process, the temperature is higher due to the sharp increase of the rotating speed. At this time, the material flow speed becomes faster, and a large amount of material in the shoulder stirring area is driven to the retreating side of the joint. A part of the material is extruded to the pin stirring area (PSZ) due to the plunging action of the shoulder in the welding process. The increase of the rotating speed leads to the strengthening of the stirring degree of the tool pin, the width of the pin driving material extends outward. Some of the heat affected zone (TMAZ) material is towed to the pin agitation zone (PSZ). The high-speed rotation of the spindle exacerbates the rotating upward flow tendency of the material in the pin stirring zone. In addition, the plastic softening degree of the material in the shoulder stirring area increases. The downward movement of the shoulder driving material is more intense, and a turbulent flow area is formed at the upper part of the advancing side. The material flow in this area is extremely intense. When the material on the retreating side cannot fill the cavity behind the tool in time, welding defects such as holes are easily formed.

The occurrence of welding defects will significantly reduce the quality of joints and even lead to the scrapping of welding materials, which will increase the cost of welding and is not conducive to the actual production demand. Hence, it is particularly important to reduce welding defects. The material flow in friction stir welding process is closely related to welding quality. The real temperature in the welding area can be approximated by temperature monitoring at the shoulder and the tool pin. The spindle speed has a significant influence on the temperature during welding process. The temperature monitoring of different welding parameters can be completed by studying the temperature distribution under different spindle speeds. In the future, a reasonable spindle speed range can be studied in combination with the welding quality analysis of welded joints. The welding parameters can be optimized by temperature monitoring. The optimized parameters will be applied to the overall welding of the fuel tank of the space rocket. The wireless temperature measurement system can be used for real-time feedback adjustment of welding equipment and real-time control of welding process parameters in the future.

## 5. Conclusions

The temperature in the welding area was monitored by a wireless temperature measuring system, the temperature distribution at different rotating speeds was studied, the flow law of materials was studied by numerical simulation, a material flow model was proposed by combining the numerical simulation results with the microstructure analysis of the weld, the material flow mode of the joint section at different rotating speeds was studied, and the formation mechanism of welding defects was studied by using the material flow model. In the future, the welding parameters can be optimized by combining the temperature testing method, which will effectively guide the improvement of welding process parameters. Based on the results of this study, the following conclusions were made:

(1) The wireless temperature measurement system can effectively capture the dynamic change of temperature in friction stir welding process. The temperature of the shoulder is always higher than that of the tool pin in the steady welding stage, and the temperature rise rate of the tool pin is higher than that of the shoulder in the initial plunging stage. However, when the shoulder contacts the material, the shoulder temperature eventually exceeds the tool pin temperature. The average temperature on the advancing side is 19 °C higher than that on the retreating side in the welding process;
(2) When the spindle speed rises from 500 rpm to 1300 rpm, the axial force decreases by 4163 N and the torque decreases by 15.2 N-m. As the temperature rises, the deformation resistance decreases, while the axial force and torque decrease. However, when the spindle speed increases from 900 rpm to 1300 rpm, the decreasing speed of the axial force and torque decreases. The validity of the finite element model is verified by comparing the experimental data with the numerical simulation data.
(3) Through the verified finite element model, the flow law of material was studied. The numerical simulation shows that the particles in the lower part of the tool pin tend to spiral upward along the tool pin. Some of the TMAZ zone particles are dragged to the PSZ zone. The shoulder has a certain plunging effect on the material, which is also the key to the formation of the weld.
(4) The effective strain on the advancing side is significantly higher than that on the retreating side. However, the effective strain reflects the action strength between the stirring tool and the material, thus indicates that the flow strength of material on the advancing side is greater than that on the retreating side.
(5) When high-rotation speeds (HRS) are chosen, the material beneath the tool shoulder tends to be extruded into the pin stirred zone (PSZ) after flowing back to the advancing side. This will cause turbulence phenomenon in the advancing side of the joint, which will easily lead to the formation of welding defects. In the future, temperature monitoring methods and material flow model can be used to optimize the welding parameters.

## Figures and Tables

**Table 1:** Thermal characteristics of the 2A14-T6 workpiece and W6 tool.

Properties	AA 2A14-T6	Tool Steel W6
Specific heat capacity (J/kg ℃)	2.46	3.18
Young’s modulus ($N/{\mathrm{mm}}^{2}$)	69,300	230,000
Heat expansion coefficient (μmm/mm/℃)	21	11.9
Thermal conductivity (N/s/℃)	176	30.8
Poisson’s ratio (/)	0.33	0.3

**Table 2:** Chemical composition of 2A14 Aluminum Alloys.

Cu	Si	Mn	Mg	Fe	Zn	Ti	Ni	Al
3.9 ~ 4.8	0.6 ~ 1.2	0.4 ~ 1.0	0.4 ~ 0.8	≤0.7	≤0.3	≤0.15	≤0.1	Margin
